# A platform for target prediction of phenotypic screening hit molecules

**DOI:** 10.1016/j.jmgm.2019.107485

**Published:** 2020-03

**Authors:** Nadine Homeyer, Ruud van Deursen, Bernardo Ochoa-Montaño, Kathrin Heikamp, Peter Ray, Fabio Zuccotto, Tom L. Blundell, Ian H. Gilbert

**Affiliations:** a Drug Discovery Unit, Division of Biological Chemistry and Drug Discovery, School of Life Sciences, University of Dundee, Sir James Black Centre, DD1 5EH, United Kingdom; b Department of Biochemistry, University of Cambridge, Old Addenbrooke’s Site, 80 Tennis Court Road, Cambridge, CB2 1GA, United Kingdom

**Keywords:** Fragment-based target prediction, Ligand similarity, Scaffold hopping, Cavity comparison, Hit docking with constraints

## Abstract

Many drug discovery programmes, particularly for infectious diseases, are conducted phenotypically. Identifying the targets of phenotypic screening hits experimentally can be complex, time-consuming, and expensive. However, it would be valuable to know what the molecular target(s) is, as knowledge of the binding pose of the hit molecule in the binding site can facilitate the compound optimisation. Furthermore, knowing the target would allow de-prioritisation of less attractive chemical series or molecular targets. To generate target-hypotheses for phenotypic active compounds, an *in silico* platform was developed that utilises both ligand and protein-structure information to generate a ranked set of predicted molecular targets. As a result of the web-based workflow the user obtains a set of 3D structures of the predicted targets with the active molecule bound. The platform was exemplified using *Mycobacterium tuberculosis,* the causative organism of tuberculosis. In a test that we performed, the platform was able to predict the targets of 60% of compounds investigated, where there was some similarity to a ligand in the protein database.

## Highlights

• An algorithm to predict the molecular target(s) of phenotypic hits against TB.
• Uses information based on the ligand and protein structure.
• Allow visualisation of proposed binding pose.
• Web interface developed.

## Graphical abstract

**Figure:**
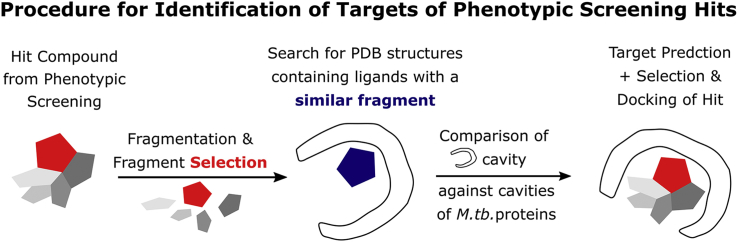

## 1 Introduction

Phenotypic drug discovery is a powerful way to conduct drug discovery programmes [1,2], particularly in the area of infectious diseases, where there are very few well-validated molecular targets. Phenotypic drug discovery does not necessarily pre-suppose a given target or pathway. The advantage of phenotypic screening is that compounds that are active in phenotypic screening modulate a mechanism(s) or pathway that is essential for the survival of the organism. In addition, these compounds have the correct properties for permeation through the cellular envelope, are metabolically stable in the infectious organism and access the molecular target(s) without significant efflux, which is a major problem in certain disease areas such as tuberculosis (TB) and Gram-negative bacteria. Due to the poor compound permeation, compound metabolism and to the presence of efflux, it can be in fact difficult to achieve the required compound exposure within a cell. Often target-based approaches fail due to low intracellular compound levels.

However, one of the major limitations of phenotypic drug discovery is lack of knowledge of the molecular target and the binding mode of the hits within the target. Such knowledge could enable a structure-guided approach leading to a focused medicinal chemistry programme. Further, many phenotypic projects are halted by issues connected with the chemical scaffold of the active series, such as poor pharmacokinetics or toxicological problems. Knowledge of the binding mode of the compound to the target protein would greatly facilitate “scaffold hopping” [3,4] to circumvent these issues. Additionally, some proteins and pathways are more attractive drug targets than others, and knowledge of the target will help to prioritise which hits should be progressed. Compounds often interact with multiple proteins [5]; knowing potential additional targets of a compound is important in compound optimisation. However, determining the targets of hit molecules from phenotypic screens experimentally can be complex, expensive, time-consuming and not always successful. Therefore, being able to predict targets computationally would be highly beneficial, as it could provide hypotheses to be tested experimentally.

As part of our effort to identify better treatments for tuberculosis we are actively involved in a number of phenotypic drug discovery projects. During the past few years we have carried out several phenotypic high throughput screening campaigns testing more than 1 million compounds and identifying multiple active chemical series. In this paper, we report the development of an *in silico* platform that is able to produce target hypotheses for phenotypic actives against *Mycobacterium tuberculosis* (*M. tb*.). The approach utilises both the structure of the active hit compound and the part of the *M. tb.* proteome for which a crystal structure is available or that can be modelled with a high degree of confidence. The 2D chemical structure of the phenotypic hit of interest is the initial input, the output is a set of ranked potential targets, and for each target, relevant binding poses are generated.

During the last 15 years, a number of computational target identification algorithms have been developed [[6], [7], [8], [9], [10], [11], [12], [13], [14], [15], [16], [17], [18], [19], [20]]. Many of them are based on the similarity of hit molecules to other compounds for which targets are known [[6], [7], [8], [9], [10], [11]]. Some algorithms take advantage of data mining or machine learning methods [7,20] to perform extensive data mining and search for compounds that are similar to the active ones [12,13,20,21]. Other approaches use the similarity in bio-activity spectra or transcriptional profiles for target prediction [14,15]. An example of a TB-specific approach is from Martínez-Jiménez et al. 2013 [22], who performed a network-based target prediction for a large set of *M. bovis* and *M. tb.* phenotypic screening hits from an analysis of GlaxoSmithKline [22]. Some methods explicitly take the 3D properties of the target into account. These can be based on large collections of pharmacophore models derived from the binding sites of known targets [16]. Alternatively reverse docking approaches have been developed where the hit molecule is docked into a large number of possible target structures [[17], [18], [19],^23^,^24^]. These 3D approaches are highly computationally demanding, and calculation runtimes can be a major bottleneck. They are also limited by the number of 3-dimensional protein structures available [18,25]. For example, in May 2017 there were 554 unique proteins from *M. tb.* in the PDB [26] which corresponds to only about 13% of the *M. tb.* proteome. Homology models have been produced of the *M. tb.* proteome; as an example the CHOPIN database [27].

The approach that we describe here was designed to generate hypotheses of potential targets of phenotypic hits and their potential binding modes within the protein, utilising the structure of both the ligand and targets.

## 2 Outline of approach

The binding of a small molecule to the site of a protein target can be seen as a molecular recognition event where the ligand will be anchored into the protein active site through key interactions between the ligand and protein, such as hydrogen bonds, dipole-dipole, π-stacking and hydrophobic interactions. These specific interactions define a molecular pharmacophore.

The premise of our approach is that structurally similar compounds interacting through a similar pharmacophore will be recognized by a similar protein binding site. Given a phenotypic hit molecule, if we could identify a related compound with a very similar pharmacophore bound to a specific protein site in the Protein Databank (PDB) [28], then we could use that information to identify in *M. tb.* any protein that has a similar binding site and further explore the binding of the phenotypic hit to that binding site to formulate target hypotheses.

Despite the increasing number of structures deposited in the PDB the number of small molecules bound to a protein binding site in the PDB covers a limited amount of chemical space (there are 26,672 small molecule ligands in the PDB, Nov 2018). This reduces the chances of identifying a small molecule ligand in the PDB that is similar to the molecular hit identified by the phenotypic screening.

The fragment-based drug discovery approaches developed in the past 20 years [29] have shown that working in a low molecular complexity space greatly increases the efficiency of the sampling of the chemical space and that fragment hits can be identified even when libraries smaller than typical HTS libraries are screened. By analogy to the fragment-based drug discovery process, we have fragmented all the small molecule ligands in the PDB and created a database capturing, for each fragment, the specific sub-pocket that recognizes the fragment itself and the specific interactions it establishes with the protein. We then reduced the molecular complexity of the phenotypic hit by fragmenting its chemical structure to generate *in silico* a set of related molecular fragments. These fragments from the phenotypic hit can then be compared to fragments from the PDB small molecule ligands.

Following an experimental screening campaign, as part of our hit evaluation process, we normally generate and test a small number of close analogues in order to gain an understanding of the relevance of the functional groups and formulate a first hypothesis of the minimum pharmacophore associated to the biological response. This knowledge can be used to select one or more *in silico* fragments generated from the initial phenotypic hit, to start the target hypothesis generation process. The first step is the identification of the fragment in the PDB that is either identical or most similar to the fragment representing the phenotypic active. This fragment will define a pocket in the PDB structure and a set of interactions that should be coherent with the initial pharmacophore.

**Fig. 1:**
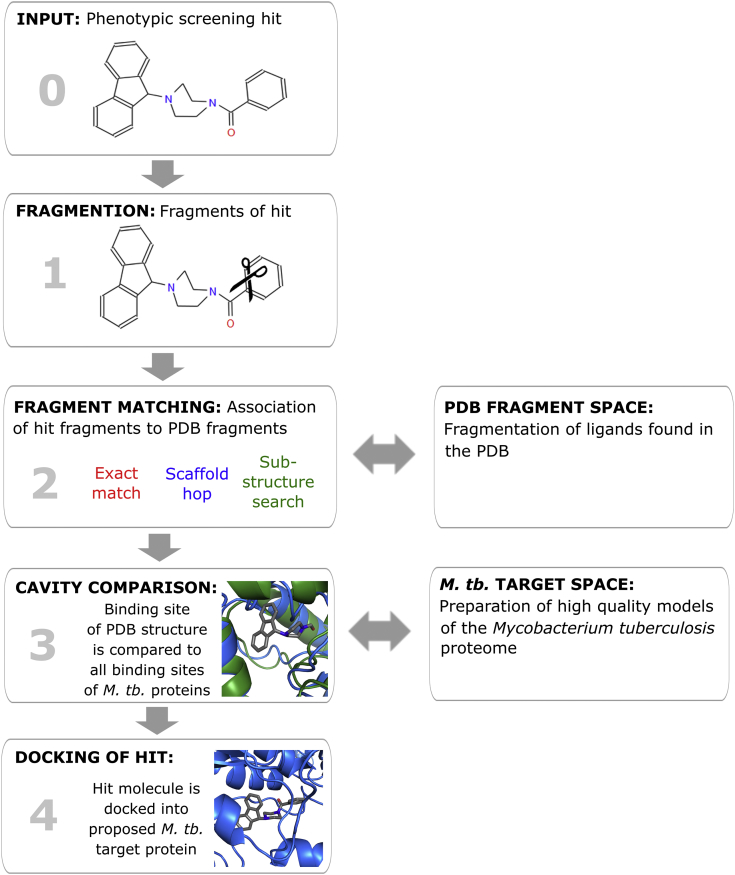
Schematic depiction of the workflow of the target prediction platform starting from the input of the hit molecule and ending with the output of the hit-target complex. The example hit molecule was taken from He et al. 2008 [30].

The next step is to see if there is a similar pocket or sub-pocket within the pathogen (*M. tb.*) proteome to that found in the PDB. This pocket or sub-pocket should bind the “fragment” of the phenotypic active in a similar manner to that observed in the PDB. As a check, we aim to refit the entire phenotypic active compound into the pocket, and to verify if this putative binding mode in the putative target can explain any observed SAR. The applicability of this approach can be limited by the fact that the PDB contains a relatively small number of crystal structures from any given pathogen (in this case *M. tb.*). To address this problem, we created a database of high confidence *M. tb.* modelled structures covering a larger portion of the *M. tb.* proteome. The overall concept is outlined in Fig. 1.

## 3 Workflow of the target prediction platform

Before starting the process, there were some key preparative steps. Firstly, the PDB ligands were fragmented to provide a database of fragments (PDB fragment space). The ligands were fragmented to generate as many molecular fragments as possible, to ensure that as many pharmacophoric patterns as possible are captured (see Supporting Information S1A). For each fragment a binding cavity is then defined and the fragment-protein interactions analyzed. We also needed to generate a *M. tb.* target space (more than 5700 structures in total) including the existing *M. tb.* structures in the PDB (2,055 structures) and a set of high-quality *M. tb.* modelled protein structures (3,667 structures), which were generated using Rosetta [31]. (see Supporting Information S2). We initially generated homology models for all the proteins encoded in the proteome, but only including in the *M. tb.* target space those models built on highly homologous template structures, which meant that not all *M. tb.* proteins were represented in our database. The Rosetta homology modelling was setup to generate homo- and hetero-oligomers whenever data were available. For each structure/model, the molecular surface was analyzed to identify potential binding cavities.

The platform workflow starts with the input of the phenotypically-active compound and consists of four main steps (Fig. 1).

**Step 1: Fragmentation of phenotypic active.** The active hit molecule is split into fragments in such a way that the pharmacophoric features of the molecule are maintained (see Supporting Information S1A). The user selects which fragments of the hit compound are used as input (fragment query). The more structure-activity relationship data that are known for the phenotypically active molecule, the better the user is able to select a fragment query that contains those chemical features that are essential for activity, to use for the database search.
**Step 2: Fragment Matching.** A search is carried out to see if the fragment query maps to the PDB fragment space. If there is no direct match, a similarity search can be conducted. Two different options are available to search for similar fragments. Firstly, an exact / similarity substructure search identifies fragments that are identical or that have a highly similar structure. Secondly, an algorithm has been developed which allows detection of fragments with a similar connectivity and element composition, but not necessarily a similar substructure (see Supporting Information S1B).
**Step 3**: **Cavity Comparison.** When an exact match or a similar fragment for the fragment query is identified in the PDB fragment space, the binding cavity for the fragment in the PDB is retrieved. The identified fragment cavity is used as query in a cavity comparison search to identify in the *M.tb.* target space structures with a similar binding pocket. *M. tb.* proteins with binding sites that are highly similar to the binding site that recognizes the query fragment in the PDB can be considered as potential targets of the phenotypically active molecule. Four different cavity comparison algorithms have been implemented in the platform: BioGPS [[32], [33], [34]], SubCav [35], FuzCav [36], aCSM [37] (see Supporting Information S2).
**Step 4:** As a validation step, the “original” phenotypically active molecule is docked into the binding site of the *M. tb.* protein identified as potential target. This step can help to identify false positives, if either the phenotypic active does not fit in the active site or the binding mode does not explain any observed SAR. Restraints can be used for the docking step to try and match the proposed binding mode and binding interactions of the phenotypic active (see Supporting Information S1D). The binding site of the hit and the docking result can be visually inspected in a molecular viewer [38]. In addition, the complex can be downloaded in PDB format and in formats optimized for the viewers, Pymol [39], Maestro [40], and ICM Browser [38].

## 4 Architecture of the target prediction platform

**Fig. 2:**
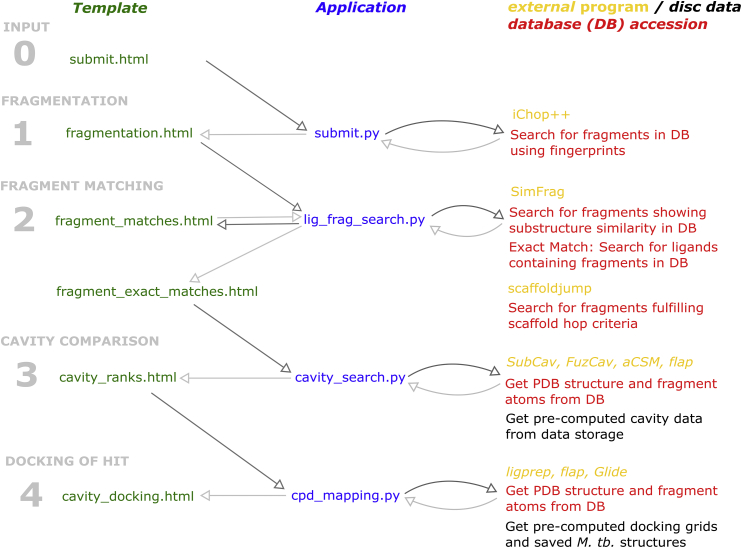
Schematic depiction of the architecture of the target identification platform. Arrows show the information workflow. External programs used within the platform are marked in orange, italic writing, whereas programs written for the purpose of the platform are given in normal font. (For interpretation of the references to colour in this figure legend, the reader is referred to the Web version of this article.)

The target prediction platform is accessible via a web-interface which is build based on the python package Flask [39] and the development server integrated therein. Template files for rendering the webpages are written in HTML (Fig. 2, green). Applications and associated scripts are written in python (Fig. 2, blue). The applications access databases created for the platform, containing information about PDB structures and the modelled *M. tb.* structures (Fig. 2, red). Other data are directly stored on disc and accessed from there (Fig. 2, black). In addition, the different programs (Fig. 2, orange) are called from the applications. Some applications were written in house, based on OpenBabel [41]: the fragmentation program *iChop++*, the similarity search program *SimFrag*, and the program *scaffoldjump* that are written in C++. Additional external programs are also in the pipeline: BioGPS [[32], [33], [34]], SubCav [35], FuzCav [36], aCSM [37], LigPrep [42], and Glide [[42], [43], [44], [45]]. The configuration of the system including the specification of database access as well as of all input and output paths is managed in a centrally stored file config.py. Therefore, the system can be easily setup in another computing environment.

## 5 The web-interface

**Fig. 3:**
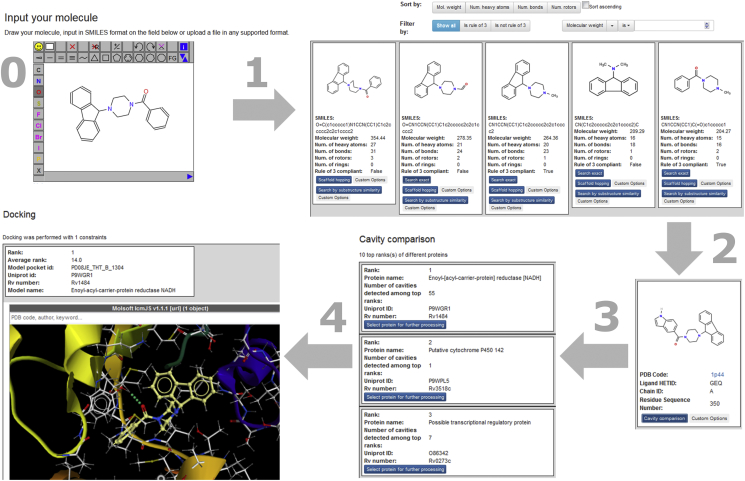
Snapshots of the web-interface showing the workflow of the platform. Numbers correspond to the numbers of the workflow steps given in Fig. 1.

In order to make the platform as user-friendly as possible, we generated a workflow that can be operated via a web-interface. It has been designed for use by medicinal chemists, who may not be experts in modelling, but can interpret both small molecule and protein structures. The front end of the applications is a set of clearly structured webpages (Fig. 3). On the first page (step 0) the user can draw or upload the phenotypically active molecule. After the fragmentation (step 1) a webpage with the fragments is shown to the user. The fragments are displayed in white rectangles within a grey box. They can be sorted by pressing the buttons “Mol. weight”, “Num. of heavy atoms”, “Num. bonds”, and “Num. rotors” above the grey box (Fig. 3, top right image). In addition, the fragments can be filtered by molecular weight. It is also possible to only show those fragments that fulfill the rule of three or that do not fulfill the rule of three by clicking on the “Is rule of 3” or “Is not rule of 3” buttons. Using this functionality, it is easy to inspect the obtained fragments and find fragments with the desired properties. The more information that is known about the SAR of the phenotypic active, the better the user is able to select the fragments for pursuing. In general, the more similar the PDB fragment is to the phenotypic active, the more predictive the binding mode is likely to be.

When a fragment has been selected, this is matched with the PDB ligand fragments (step 2). The PDB structures containing this fragment are displayed. A link to the Protein Data Bank [26] enables an easy inspection of the PDB structures that are found. When one of the structures is selected, the cavity comparison is carried out (step 3). The result of this step is a webpage listing those ten proteins that possess the most similar binding sites to the binding site of the selected PDB structure. These proteins can be filtered by essentiality for M. tb. survival according to DeJesus et al., 2017 [46] and Griffin et al., 2011 [47]. After selection of one of the protein structures, the hit molecule is docked into this potential target structure (step 4). On the results page of the docking step, the generated complex structure is graphically displayed in a molecular viewer. The viewer is the IcmJS [38] Java Script applet that provides beside a number of viewing options, also possibilities to manipulate the molecules and to generate slides. The functionality of IcmJS [38] is very similar to that available in the ICM Browser [38] and ICM-Pro [48] tools used by many chemists. In addition to the visual output, files for download are provided in many common formats so that the users can visualize the results in their favorable visualisation tool.

The possibility to operate the backend workflow via a front-end web-interface ensures that the system is also accessible to users with only limited computational experience.

## 6 Case study

As a case study we analyzed ligands annotated in the TIBLE database [49] as binders of *M. tb.* proteins. The TIBLE database contains besides minimal inhibitory concentrations for compounds against *Mycobacterial* species, also binding data for specific protein targets for *M. tb.* [49]. For each *M. tb.* target protein the ligand, with the lowest annotated (i.e. most potent) IC_50_, K_i_, or K_d_, was selected. For some of the 106 protein targets in TIBLE [49] no ligands with IC_50_, K_i_, or K_d_ were available in the database. In total 48 protein – ligand interactions were retrieved (see Supporting Information S5, carried out in 2017). SMILES of the ligands were obtained from ChEMBL [50] and used as input for the target identification platform. A complete analysis workflow was carried out, wherever possible, using the default settings.

## 7 Results

It is challenging to carry out a rigorous evaluation of the algorithm. However, we chose to use examples listed in the TIBLE database. There is enzyme inhibition data for compounds against different enzymes in the TIBLE database. However, it is not known if these compounds are active phenotypically against *M. tb.* and if so, whether the phenotypic activity of the compounds against *M. tb.* (if any) is due to inhibition of this enzyme. The enzyme activity data will also be dependent on the conditions under which it was measured. Nonetheless, despite these caveats, it provides a database against which to test the algorithm. For the 48 ligands that were retrieved from the TIBLE database [49], a target prediction analysis was performed. No target could be predicted in 16 cases, because there were no ligands or fragments present in the PDB database that were sufficiently similar to the analyzed TIBLE ligands. In such cases the analysis was terminated at the “Fragmentation” or “Fragment matching” steps (Fig. 1). These cases were not included in the evaluation of the platform, because in a real target search scenario a user would have identified the problem and would not have made a target prediction based on the very dissimilar ligands.

The remaining 32 cases were considered for performance analysis. A prediction was counted as correct, when the target annotated in TIBLE was among the top 10 predicted targets or among the 10 targets listed after filtering to remove non-essential proteins as defined by DeJesus et al., 2017 [46]. It must be noted that the algorithm may have also identified other proteins to which the phenotypic actives bind, but which have not (yet) been identified as binding partners through experimentation and that these other molecular targets could be responsible for at least some of the phenotypic activity.

**Fig. 4:**
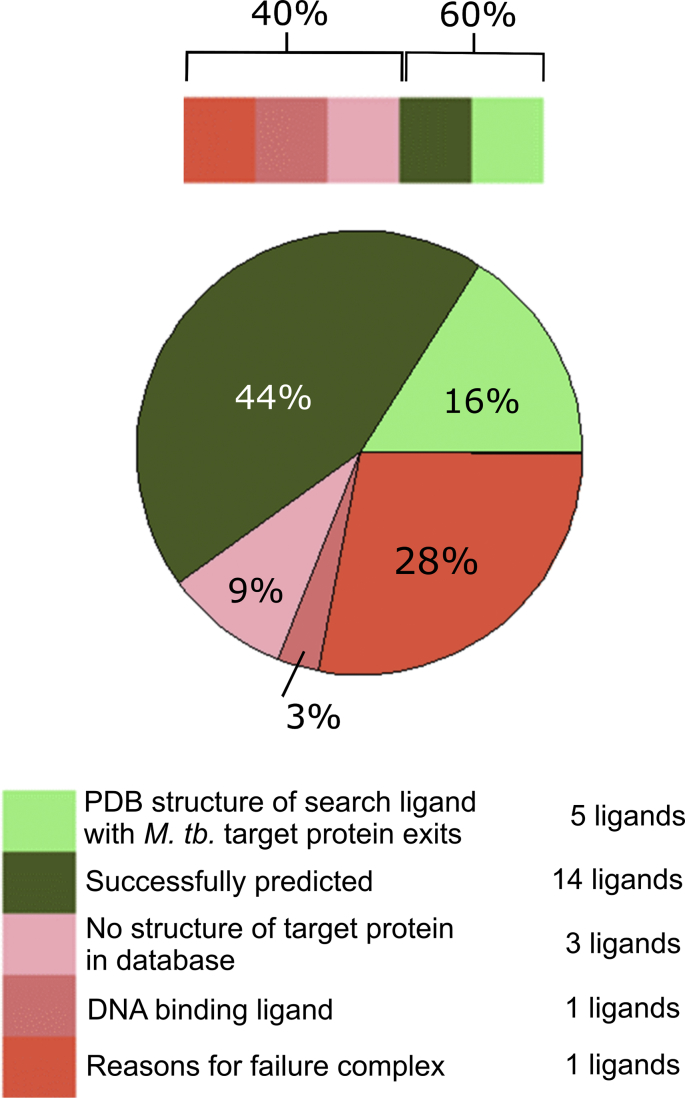
Pie chart of the results of the case study analysis for 32 ligand – target protein associations from the TIBLE database. In addition to the targets from the TIBLE database the platform can predict other, so far not experimentally identified targets that might be responsible or add to the phenotypic effect. If these targets could be taken into account, it could increase the hit-rate of the algorithim.

Out of the 32 cases, a correct prediction was possible for five ligands, where the ligand and/or the protein was not highly similar to the ligand and/or protein from TIBLE (Fig. 4). Proteins were not regarded as similar when the PDB protein was different from the target protein or when the PDB protein was the same protein as the target protein but from a distantly related organism. In addition to these challenging predictions, there were nine cases where ligands and/or proteins were similar to the ligands/proteins from TIBLE. With respect to the protein this meant that the PDB protein was the same protein as the target protein, but from a closely related organism. Ligands in this category contained, for example, the same core as the TIBLE ligand, however, had additional substituent(s) or similar but different functional groups. Beside these successful target predictions there were five cases where a crystal structure of the target protein in complex with the analyzed ligand existed. In all of these cases the “self-search”, that is the search of the target structure based on the same protein-ligand complex structure from the PDB, was successful. In total the percentage of the 19 successful cases sums up to 60%.

There were 13 cases where the platform failed to provide a correct prediction (although of course there could be other targets that the algorithm predicted, but have not yet been identified experimentally). For three of the studied targets there were no PDB or model structures in the *in-house M. tb.* structure database. Such cases cannot be recognized by the user, but cannot be prevented either. The database currently covers ∼40% of the *M. tb.* proteome and will be extended as much as possible as more and more PDB structures become available with sufficient sequence similarity. However, we decided that we would only include modelled *M. tb*. structures where there is a high degree of confidence.

In one case (CHEMBL1818383, Rv0467) the prediction was not successful because the ligand in the identified PDB template structure (PDB code: 5bta) binds at the interface to the DNA. As the currently implemented cavity comparison methods cannot take nucleotide molecules into account such cases cannot be handled by the system. A user can identify such cases by looking at the binding site of the ligand in the identified PDB structure. A direct link to the respective PDB webpage facilitates a visual inspection of the binding position of the ligand.

In nine cases the target could not be predicted due to reasons that are not obvious. There can be explanations for some of these failures (see discussion), but the user of the platform would most of the time not be able to identify these, because internal knowledge of the database content is required. Therefore, these nine cases in addition to the three cases where the target protein is not in the *M. tb.* structure database need to be considered as unsuccessful predictions.

A further complication is that often inhibitors bind to multiple proteins. So whilst the compounds may not be predicted to bind to the protein highlighted in TIBLE, it is conceivable that the compounds may bind to other proteins in addition to those indicated in TIBLE. Further, the phenotypic response of the pathogen may or may not be related to inhibition of a particular enzyme.

## 8 Discussion

The target prediction platform presented in this manuscript has proven to be capable of predicting the targets of ligands of *M. tb.* proteins for which binding affinity data are available [49]. Even if those cases where the PDB *M. tb.* crystal structures for the respective protein-ligand exist, are not considered, the prediction was successful in 44% of the cases. On the other hand there are 37.5% of cases for which the prediction was not successful. Among these, 9% (3 test cases) failed due to the absence of the protein target in the database. As more and more PDB structures become available and new *M. tb.* models are built based on them, it can be expected that the number of such cases would decrease.

Among the ligands for which targets were successfully predicted, were ligands of well-known and largely studied *M. tb.* targets as PknB (Rv0014c), peptide deformylase (Rv0429c), Cyp51 (Rv0764c), InhA (Rv1484), ftsZ (Rv2130c), KasB (Rv2246), cysK1 (Rv2334), aroQ (Rv2537c), tmk (Rv3247c), and the reductoisomerase dxr (Rv2870c), as well as the thymidylate synthases ThyA and ThyX (Rv2764c, Rv2754c) but also ligands of relatively unexplored targets such as the NAD kinase (Rv1695).

In 28% of the cases the target prediction was not successful for reasons that were not obvious. The causes for the inability of the algorithm to predict the expected targets can be complex. While the binding affinity data in the TIBLE database prove that the ligands are targeting the respective proteins, it is not known whether the annotated interacting protein is the primary target of the ligand compound.

**Fig. 5:**
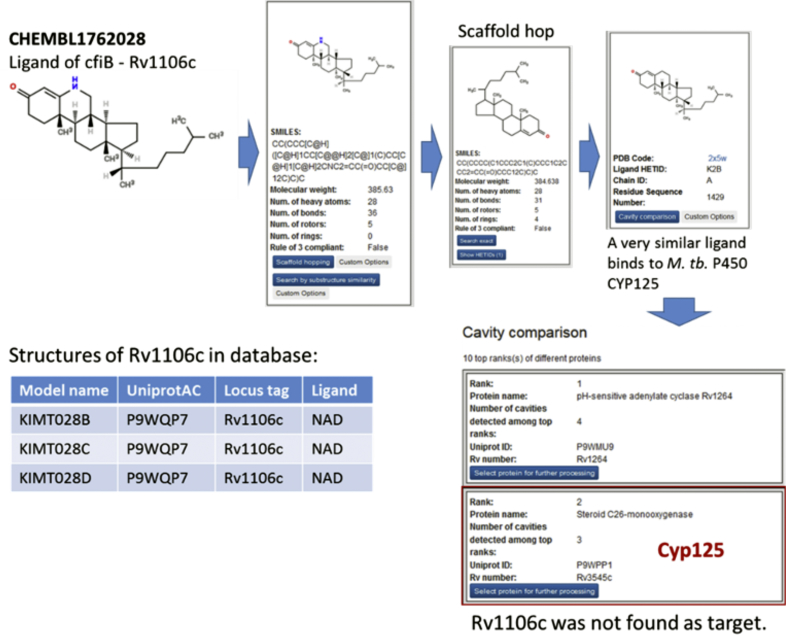
Target search for CHEMBL1762028 (ligand of Rv1106c, cifB). Workflow on top and on the right side shows the analysis steps. The table on the bottom left lists the structures that are available in the database.

For example, for CHEMBL1762028 the target Rv1106c (cifB) annotated in TIBLE was not found (Fig. 5). However, the platform identified *M. tb.* Cyp125 (Rv3545, PDB entry: 2x5w) as a potential target. Indeed, there is a very similar ligand to CHEMBL1762028 bound to this target in the PDB. Therefore, Cyp125 is likely an alternative target of CHEMBL1762028. The identification of Rv1106c is likely hampered by the absence of structures of the cifB protein in complex with a similar ligand. The database comprises three model structures of cifB, all of which contain NAD as bound ligand. As the CHEMBL ligand has no similarity with NAD, it likely binds at a different binding site that is not annotated in the system and could therefore not be identified in the cavity comparison.

In the case of CHEMBL1446150, the target annotated in TIBLE was Rv1284, a β-carbonic anhydrase. This target was not identified in our algorithm. However, a *M. tb.* crystal structure of the O-phosphoserine sulfhydrylase (Rv1336) in complex with a ligand similar to the CHEMBL compound was found. There are four PDB structures and one modelled structure of Rv1284 in the *M. tb.* structure database. All of these are apo structures, where the annotated binding sites are less certain.

A case where the reason for the failure is less clear is CHEMBL608841, which is annotated as inhibiting salicyl-AMP ligase (Rv2384, MbtA) in TIBLE. Here a crystal complex structure of the peptide arylation enzyme of *Acinetobacter baumannii* (PDB-entry: 3o83) was identified as template for cavity comparison. Both the 3D structure of the protein and the bound ligand are similar to the target models and the ChEMBL ligand respectively. The model structures of the target (Rv2384) in the *M. tb.* structure database, shows a sufficiently large 3D similarity with the PDB template structure, and seven of them have similar ligands bound to the same binding site as the PDB template protein. It is therefore not obvious why Rv2384 is not found as a potential target. One reason for this could be that the aliphatic side chain moiety of the ligand in the template PDB structure (PDB-entry: 3o83), that is absent in the ligands of the model *M. tb.* structures, prevents a correct detection of the binding site of the target protein. A correct identification of the target might also be hampered by the fact that the PDB template structure does not cover the whole sequence of the peptide arylation enzyme of *A. baumannii*, so that the C-terminus adopts a structure in which it comes in close contact with the binding site. In line with this the results indicate that most likely the differences between the binding sites of the PDB and the target structures are still too large to detect the similarity and the target binding site is lost in the noise especially from unspecific apo pocket binding sites.

Consistent with our expectations we generally have observed that apo pockets are relatively seldom identified as correct target binding sites and seem rather to contribute noise to the cavity comparison step. This is probably the case, because the whole pockets and not only the regions where a ligand would potentially bind have been used for the definition of the binding sites.

Despite the different levels of data certainty and some minor drawbacks (such as non-protein targets, proteins for which a reliable homology model could not be prepared), the platform has overall proven to be capable to predict the targets of ligands for which binding data were available in the TIBLE database with a reasonable success rate (see Fig. 4). It can therefore be expected that the platform is also able to be useful in the prediction of the targets of phenotypically active compounds. A great advantage of the platform is that the associated data and the backend database can easily be updated, so that it will be possible to keep the system up to date and consider the large amount of additional PDB structures that become newly available each month. In the future we plan to improve some aspects of the platform as the apo pocket definition and the chemical space available for fragment matching.

## 9 Conclusions

We have developed a computational method for predicting the targets of phenotypic hits in *M. tb*. On test cases, the algorithm has a good success rate in predicting the target of phenotypic hits. We see this as a hypothesis generating tool, which needs to be followed up experimentally. There are several factors to bear in mind here. There are some drugs which do not target proteins, and these will not be identified by this approach. Many drugs actually bind to multiple proteins, some of which, but not necessarily all, are responsible for the phenotypic effect. Some or all of these will be predicted by the algorithm. Therefore, in our test data, some predicted targets may look like false positives, but actually may be binding partners. A limitation of the algorithm is the dependence on high quality models of the *M. tb.* proteins. This will expand over time. However, certain protein classes are not well represented, such as membrane proteins. Another challenge is that the chemical space of the PDB ligands does not reflect all of the space of the phenotypic hits. Despite all these caveats, the algorithm is remarkably good at identifying potential drug targets. A further benefit of the algorithm is that it also can predict a binding mode of the molecule in the target, which has great value in compound optimisation. However, as with all modelling, it is important that the user recognizes that this platform is hypothesis generating and needs to be confirmed experimentally.

Much drug discovery for infectious diseases is carried out using phenotypic screening, owing to the lack of highly validated drug targets. The lack of highly validated targets is in part due to an incomplete understanding of the biology of the pathogens; what are the physiologically relevant key pinch points. In many cases it is not known whether it is possible to inhibit an enzyme with a compound that has the correct (oral) drug-like properties. Other factors are also important such as the rate of kill of the organism and the degree (percentage) by which a target must be inhibited to have a pharmacological effect. As discussed above, in some cases there are also issues such as compound penetration, metabolism and efflux.

The work reported here has focused on developing the platform for tuberculosis. However this could be extended to other pathogens, by changing the database of proteins. This would require extracting protein structures from the protein databank, for the pathogen of interest, and making high quality models where there were no experimental structures.

## Declaration of competing interest

The authors declare no conflicts of interest.

## References

[1] Swinney D.C., Anthony J. (2011). How were new medicines discovered?. Nat. Rev. Drug Discov..

[2] Gilbert I.H. (2013). Drug discovery for neglected diseases: molecular target-based and phenotypic approaches. J. Med. Chem..

[3] Bajorath J. (2017). Computational scaffold hopping: cornerstone for the future of drug design?. Future Med. Chem..

[4] Hu Y., Stumpfe D., Bajorath J. (2017). Recent advances in scaffold hopping. J. Med. Chem..

[5] Mestres J., Gregori-Puigjane E., Valverde S., Sole R.V. (2009). The topology of drug-target interaction networks: implicit dependence on drug properties and target families. Mol. Biosyst..

[6] Gfeller D., Grosdidier A., Wirth M., Daina A., Michielin O., Zoete V. (2014). SwissTargetPrediction: a web server for target prediction of bioactive small molecules. Nucleic Acids Res..

[7] Wang L., Ma C., Wipf P., Liu H., Su W., Xie X.Q. (2013). TargetHunter: an in silico target identification tool for predicting therapeutic potential of small organic molecules based on chemogenomic database. AAPS J..

[8] Keiser M.J., Setola V., Irwin J.J., Laggner C., Abbas A.I., Hufeisen S.J., Jensen N.H., Kuijer M.B., Matos R.C., Tran T.B., Whaley R., Glennon R.A., Hert J., Thomas K.L., Edwards D.D., Shoichet B.K., Roth B.L. (2009). Predicting new molecular targets for known drugs. Nature.

[9] Awale M., Reymond J.L. (2017). The polypharmacology browser: a web-based multi-fingerprint target prediction tool using ChEMBL bioactivity data. J. Cheminf..

[10] Gong J., Cai C., Liu X., Ku X., Jiang H., Gao D., Li H. (2013). ChemMapper: a versatile web server for exploring pharmacology and chemical structure association based on molecular 3D similarity method. Bioinformatics.

[11] Nickel J., Gohlke B.O., Erehman J., Banerjee P., Rong W.W., Goede A., Dunkel M., Preissner R. (2014). SuperPred: update on drug classification and target prediction. Nucleic Acids Res..

[12] Huang T., Mi H., Lin C.Y., Zhao L., Zhong L.L., Liu F.B., Zhang G., Lu A.P., Bian Z.X., for M.G. (2017). MOST: most-similar ligand based approach to target prediction. BMC Bioinf..

[13] Liu X., Vogt I., Haque T., Campillos M. (2013). HitPick: a web server for hit identification and target prediction of chemical screenings. Bioinformatics.

[14] Cheng T., Li Q., Wang Y., Bryant S.H. (2011). Identifying compound-target associations by combining bioactivity profile similarity search and public databases mining. J. Chem. Inf. Model..

[15] Iskar M., Zeller G., Blattmann P., Campillos M., Kuhn M., Kaminska K.H., Runz H., Gavin A.C., Pepperkok R., van Noort V., Bork P. (2013). Characterization of drug-induced transcriptional modules: towards drug repositioning and functional understanding. Mol. Syst. Biol..

[16] Wang X., Shen Y., Wang S., Li S., Zhang W., Liu X., Lai L., Pei J., Li H. (2017). PharmMapper 2017 update: a web server for potential drug target identification with a comprehensive target pharmacophore database. Nucleic Acids Res..

[17] Li H., Gao Z., Kang L., Zhang H., Yang K., Yu K., Luo X., Zhu W., Chen K., Shen J., Wang X., Jiang H. (2006). TarFisDock: a web server for identifying drug targets with docking approach. Nucleic Acids Res..

[18] LaBute M.X., Zhang X., Lenderman J., Bennion B.J., Wong S.E., Lightstone F.C. (2014). Adverse drug reaction prediction using scores produced by large-scale drug-protein target docking on high-performance computing machines. PLoS One.

[19] Wang J.C., Chu P.Y., Chen C.M., Lin J.H. (2012). idTarget: a web server for identifying protein targets of small chemical molecules with robust scoring functions and a divide-and-conquer docking approach. Nucleic Acids Res..

[20] Ding H., Takigawa I., Mamitsuka H., Zhu S. (2014). Similarity-based machine learning methods for predicting drug-target interactions: a brief review. Briefings Bioinf..

[21] Nidhi M. Glick, Davies J.W., Jenkins J.L. (2006). Prediction of biological targets for compounds using multiple-category Bayesian models trained on chemogenomics databases. J. Chem. Inf. Model..

[22] Martinez-Jimenez F., Papadatos G., Yang L., Wallace I.M., Kumar V., Pieper U., Sali A., Brown J.R., Overington J.P., Marti-Renom M.A. (2013). Target prediction for an open access set of compounds active against Mycobacterium tuberculosis. PLoS Comput. Biol..

[23] Chen Y.Z., Zhi D.G. (2001). Ligand-protein inverse docking and its potential use in the computer search of protein targets of a small molecule. Proteins.

[24] Paul N., Kellenberger E., Bret G., Muller P., Rognan D. (2004). Recovering the true targets of specific ligands by virtual screening of the protein data bank. Proteins.

[25] Gao Z., Li H., Zhang H., Liu X., Kang L., Luo X., Zhu W., Chen K., Wang X., Jiang H. (2008). PDTD: a web-accessible protein database for drug target identification. BMC Bioinf..

[26] Berman H.M., Westbrook J., Feng Z., Gilliland G., Bhat T.N., Weissig H., Shindyalov I.N., Bourne P.E. (2000). The protein data bank. Nucleic Acids Res..

[27] Ochoa-Montano B., Mohan N., Blundell T.L. (2015). CHOPIN: a Web Resource for the Structural and Functional Proteome of Mycobacterium tuberculosis. Database. 2015.

[28] Berman H.M., Westbrook J., Feng Z., Gilliland G., Bhat T.N., Weissig H., Shindyalov I.N., Bourne P.E. (2000). The protein data bank. Nucleic Acids Res..

[29] Blundell T.L., Jhoti H., Abell C. (2002). High-Throughput crystallography for lead discovery in drug design. Nature Reviews Drug Discovery. Nat Rev Drug Discov..

[30] He X., Alian A., Ortiz de Montellano P.R. (2007). Inhibition of the Mycobacterium tuberculosis enoyl acyl carrier protein reductase InhA by arylamides. Bioorg. Med. Chem..

[31] https://www.rosettacommons.org.

[32] Siragusa L., Cross S., Baroni M., Goracci L., Cruciani G. (2015). BioGPS: navigating biological space to predict polypharmacology, off-targeting, and selectivity. Proteins.

[33] Cross S., Baroni M., Carosati E., Benedetti P., Clementi S. (2010). FLAP: GRID molecular interaction fields in virtual screening. Validation using the DUD data set. J. Chem. Inf. Model..

[34] Ferrario V., Siragusa L., Ebert C., Baroni M., Foscato M., Cruciani G., Gardossi L. (2014). BioGPS: descriptors for rational engineering of enzyme promiscuity and structure based bioinformatic analysis. PLoS One.

[35] Kalliokoski T., Olsson T.S., Vulpetti A. (2013). Subpocket analysis method for fragment-based drug discovery. J. Chem. Inf. Model..

[36] Weill N., Rognan D. (2010). Alignment-free ultra-high-throughput comparison of druggable protein-ligand binding sites. J. Chem. Inf. Model..

[37] Pires D.E., de Melo-Minardi R.C., da Silveira C.H., Campos F.F., Meira W. (2013). aCSM: noise-free graph-based signatures to large-scale receptor-based ligand prediction. Bioinformatics.

[38] Molsoft L.L.C. (2017). ICM Browser.

[39] Ronacher A. (2010-2017). Flask, Web Development, One Drop at a Time.

[40] (2017). Schrödinger Release 2017-2: MS Jaguar.

[41] O’Boyle N.M., Banck M., James C.A., Morley C., Vandermeersch T., Hutchison G.R. (2011). Open Babel: an open chemical toolbox. J. Cheminf..

[42] Halgren T.A., Murphy R.B., Friesner R.A., Beard H.S., Frye L.L., Pollard W.T., Banks J.L. (2004). Glide: a new approach for rapid, accurate docking and scoring. 2. Enrichment factors in database screening. J. Med. Chem..

[43] (2016). Schrödinger Release 2016-4: Glide.

[44] Friesner R.A., Murphy R.B., Repasky M.P., Frye L.L., Greenwood J.R., Halgren T.A., Sanschagrin P.C., Mainz D.T. (2006). Extra precision glide: docking and scoring incorporating a model of hydrophobic enclosure for protein-ligand complexes. J. Med. Chem..

[45] Friesner R.A., Banks J.L., Murphy R.B., Halgren T.A., Klicic J.J., Mainz D.T., Repasky M.P., Knoll E.H., Shelley M., Perry J.K., Shaw D.E., Francis P., Shenkin P.S. (2004). Glide: a new approach for rapid, accurate docking and scoring. 1. Method and assessment of docking accuracy. J. Med. Chem..

[46] DeJesus M.A., Gerrick E.R., Xu W., Park S.W., Long J.E., Boutte C.C., Rubin E.J., Schnappinger D., Ehrt S., Fortune S.M., Sassetti C.M., Ioerger T.R. (2017). Comprehensive essentiality analysis of the Mycobacterium tuberculosis genome via saturating transposon mutagenesis. mBio.

[47] Griffin J.E., Gawronski J.D., Dejesus M.A., Ioerger T.R., Akerley B.J., Sassetti C.M. (2011). High-resolution phenotypic profiling defines genes essential for mycobacterial growth and cholesterol catabolism. PLoS Pathog..

[48] Molsoft L.L.C. (2017). ICM-pro.

[49] Malhotra S., Mugumbate G., Blundell T.L., Higueruelo A.P. (2017). TIBLE: a Web-Based, Freely Accessible Resource for Small-Molecule Binding Data for Mycobacterial Species.

[50] Bento A.P., Gaulton A., Hersey A., Bellis L.J., Chambers J., Davies M., Kruger F.A., Light Y., Mak L., McGlinchey S., Nowotka M., Papadatos G., Santos R., Overington J.P. (2014). The ChEMBL bioactivity database: an update. Nucleic Acids Res..

