# Supplementary material for: A platform for target prediction of phenotypic screening hit molecules
Source: J Mol Graph Model. 2020 Mar;95:107485. doi: 10.1016/j.jmgm.2019.107485 (PMC6983931; doi:10.1016/j.jmgm.2019.107485)
Supplement: Multimedia component 1 [file mmc1.pdf]

Supporting Information

A platform for target prediction of phenotypic screening hit molecules

Nadine Homeyer,[1] Ruud van Deursen,[1] Bernardo Ochoa-Montaño,[2] Kathrin Heikamp,[1] Fabio Zuccotto,[1] Tom L. Blundell,[2] Ian H. Gilbert[1,*]

1. Drug Discovery Unit, Division of Biological Chemistry and Drug Discovery, School of Life Sciences, University of Dundee, Sir James Black Centre, DD1 5EH, United Kingdom.

2. Department of Biochemistry, University of Cambridge, Old Addenbrooke's Site, 80 Tennis Court Road, Cambridge, CB2 1GA, United Kingdom.

* i.h.gilbert@dundee.ac.uk

S1. Algorithms

A. Step1: Fragmentation

The phenotypically active molecule provided by the user is fragmented by the fragmentation program *iChop++*. The program is designed to generate as many molecular fragments as possible. It is important that during the fragmentation process, fragments that include the key pharmacophoric recognition motifs between the phenotypic hit and protein are generated. As all possible fragments of a molecule are generated, the resulting fragments do not necessarily conform to the rule of 3 [1].

In the first step of the fragmentation algorithm *iChop++* the bonds of the ligand molecules are ordered by increasing complexity and functionality. The bonds are then selected and cut in the pre-determined order, applying one of the following “cut modes”: a “plain cut” where the bonds are simply split; and a “respect substitution” cut where the atoms linked by the bond are kept in the generated fragments in order to maintain important pharmacophoric features of the compound (Figure S1A). Which “cut mode” is used, is determined by an extensive set of rules that is coded in the program and can be modified.

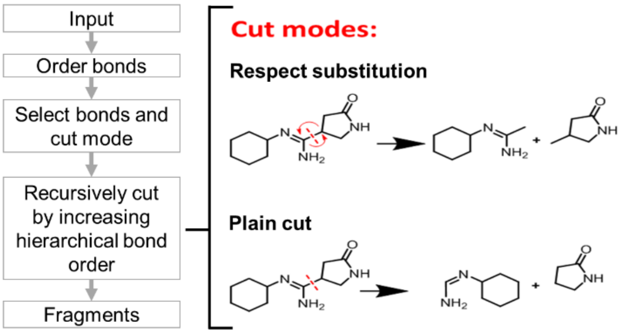

Figure S1. Schema of the fragmentation process (the first step in the target prediction workflow).

B. Step 2: Fragment matching

All fragments of the hit molecule that are generated by iChop++ in the fragmentation step can be compared to fragments of PDB ligands. If the user has an understanding of the SAR of the active series, this can help in the selection of the fragment(s) used as input for

searching. The more the input fragment(s) captures the pharmacophore associated to the original hit, the greater the chance of identifying the binding site and the less false positives.

The search for similar fragments in the PDB can be carried out using two different approaches: (A) Exact match/Substructure similarity and (B) Scaffold hopping.

A. Exact match/ Substructure similarity search

The fragments selected by the user are cross checked with the fragmented PDB ligands, to see if they are present in the PDB ligands. The comparison is performed based on OpenBabel [2] FP2 fingerprints which are generated on the fly for the fragments of the phenotypic actives and compared to fingerprints pre-computed for the PDB fragments. The fingerprints represent the substructure of the fragments, so that a 100 % match of the fingerprints indicates fragment identity. If there is an exact match, the user can directly look at the corresponding PDB structures that contains the fragment.

In the case where no fragments of the phenotypic active are found that exactly match a fragment of the PDB ligands, a substructure search can be performed. This search looks for PDB fragments that have a 2D structure that is similar to that of the hit fragment. In order to speed up the comparison of the fingerprint of the fragment of the phenotypic active with the fingerprints of all the PDB fragments, an enrichment procedure based on eight partial sums is applied.

In order to speed up the comparison of the fingerprint of the fragment of the phenotypic active with the fingerprints of all the PDB fragments, an enrichment procedure based on eight partial sums is applied. The partial sums are used to estimate the best possible Tanimoto coefficient between two 512 bit fingerprints, with the aim of finding the best entries as quickly as possible when sorted by decreasing estimated value. The Tanimoto similarity coefficient calculates the similarity of two fingerprints A and B of equal length, by dividing the number of bits that are set to 1 in both fingerprints (AND) by the number of bits set in one of

the fingerprints (OR). Therefore the best achievable Tanimoto coefficient for a single sum can be calculated as:

1. Let a be the number of bits set in fingerprint A, and b in B.
2. Then the maximum number of bits that are set in both fingerprints $MAX(AND)$ equals $MIN(a,b)$. And the minimum number of bits in one of the fingerprints $MIN(OR)$ equals $MAX(a,b)$.
3. Consequently the best Tanimoto coefficient ($T_{1sum,best}$) that can be achieved is:

$$T_{1sum,best} = MAX(AND)/MIN(OR) \qquad (1)$$

If now instead of a single sum eight partial sums are considered, the best-achievable Tanimoto coefficient ($T_{8sum,best}$) can be calculated as:

1. For each of the partial sums i=1,2,…,8:

$$MAX(AND)_i = MIN(a_i,b_i) \qquad (2)$$

$$MIN(OR)_i = MAX(a_i,b_i) \qquad (3)$$

2. Summation:

$$MAX(AND) = \mathrm{SUM}(MAX(AND)_i) \qquad (4)$$

$$MIN(OR) = \mathrm{SUM}(MIN(OR)_i) \qquad (5)$$

3. Best-achievable Tanimoto ($T_{8sum,best}$):

$$T_{8sum,best} = MAX(AND)/MIN(OR) \qquad (6)$$

For a certain selected hit fragment, $T_{8sum,best}$ is calculated for every PDB ligand fragment. The resulting fragment-$T_{8sum,best}$ list is then sorted in descending order on the estimated $T_{8sum,best}$ value. It can be expected that the candidates with the best "real" Tanimoto coefficient enrich at the top of the list. However, as the position of the bits that are switched to "on" is not considered in the calculation of the eight partial sums, the "real" Tanimoto needs still to be calculated. This is done starting from the top of the sorted fragment-$T_{8sum,best}$

list until either the number of requested maximum fragments is reached or $T_{8sum,best}$ < requested Tanimoto limit.

Fingerprints and their eight partial sums have been pre-calculated for all PDB ligand fragments using the C++ program *SimFrag* and are stored in a database. For each hit fragment an ordered fragment-$T_{8sum,best}$ list is generated from the PDB fragment data by a MySQL procedure. Afterwards, the "real" Tanimoto coefficient is calculated and a filtered list of fragments with high substructure similarity is generated by the program SimFrag.

B. Scaffold hopping

If there is no similar substructure, a scaffold hopping procedure can be used to search for fragments with similar elemental composition and connectivity. Scaffold hopping was conducted using an algorithm adapted from Ngyuen et al. [3]. For this the atom connectivity and element composition of the fragment are coded in a 63-dimensional integer fingerprint vector. Examples of the type of information about the fragment stored in the vector include: how many cyclic carbon atoms are bound to three atoms in the fragment; or the number of hydrogen atoms in the fragment that are connected to an oxygen atom. In addition to the simple connectivity information, the bond order is also coded separately in the fingerprint, such that the saturation level is also considered. The fingerprint vector of the fragment of the phenotypic active is compared to fingerprint vectors of all fragments of PDB ligands using the Manhattan distance[4] as similarity criterion. As the overall structure of the fragments is not coded in the fingerprints, PDB fragments with a similar connectivity and element composition but with a different sub-structure can be found. The algorithm for performing scaffold hops is encoded in the C++ program *scaffoldjump* (Fig. 2). This program takes as input a SMILES string of the fragment of the phenotypic active and a file containing the fingerprint vectors of all PDB fragments. An upper limit for the Manhattan distance and the maximum number of output fragments is defined. The program then creates the fingerprint for the fragment of the phenotypic active and performs a similarity comparison against all PDB fragments. The output is a list of identified similar PDB fragments and corresponding calculated Manhattan distances.

C. Step 3: Cavity comparison

The next step is to see if there is a similar binding pocket in the *M. tb.* proteome. The *M. tb.* proteome contains ~4033 proteins, of which there are structures of ~554 in the PDB (May 2017). By extensive modelling using exclusively high sequence identity, high-quality templates we were able to cover in total 1602 proteins, which is ~40 % of the *M. tb.* proteome. Modelling structures using templates with low sequence identity was considered not beneficial as it would only increase the noise in the data without any gain in prediction quality. As during the last decade, the number of *M. tb.* structures available in the PDB more than doubled [5-7], it can be expected that a much larger number of *M. tb*. protein structures will be solved, improving further the predictive power of our platform.

A cavity comparison is then conducted to see if there is a similar binding pocket in the *M. tb.* proteome to that observed between the PDB ligand fragment and its protein structure. The selected binding site is used as template in a binding site / cavity comparison against a set of 11,206 binding sites of *M. tb.* structures.

The following cavity comparison methods have been integrated into the platform and can be chosen by the user:

- SubCav, Kalliokoski *et al.* 2013 [8]: A binding site comparison method that is based on pharmacophoric fingerprints considering eight atom type associated features and the lengths of the edges of triangles between them.
- FuzCav, Weill and Rognan 2009 [9]: An alignment free binding site comparison using pharmacophoric fingerprints that take into account six amino acid features assigned to Cα atoms and the inter-Cα distances of feature triplets.
- aCSM, Pires *et al.* 2013 [10]: A binding site comparison method that is based on atomic distance signatures using either three or eight categories of atomic properties, as polar-polar and hydrophobic-polar, for distance measurements and applying a noise- and dimensionality-reduction.
- BioGPS, Siragusa et al. 2015 [11,12]: A binding site comparison method that is based on GRID molecular interaction fields (MIFs) created employing four probes. The fields

reflect the shape of the cavity (H probe), hydrophobic features (DRY probe), hydrogen bond donor properties (O probe), and hydrogen bond acceptor properties (N1 probe). Representative MIF points are selected and converted to "quadruplet points" that are then coded in fingerprints used for cavity comparison.

While all binding site comparison methods, can be run individually, it is also possible to do a consensus analysis. In this case the ranks on which a protein binding site is found using the different methods is summed up and then divided by the number of methods considered. The binding sites are then ordered by the calculated consensus rank. Protein binding sites that have a high rank in all methods will appear among the top ranks in the consensus list.

In the normal cavity comparison mode, the PDB binding site used as template for comparison is defined based on the bound ligand. All atoms that are within a distance of 5 Å (SubCav, FuzCav, and aCSM) around the ligand or 2 Å (BioGPS) around the ligand centered grid are considered for comparison.

In addition, there exists an option for a BioGPS based cavity comparison where the cavity is defined based on the fragment of the PDB ligand identified in step 2. As a fragment is usually involved in relatively few specific interactions with the protein target, compared to a typical hit from a high throughput screen, the cavity comparison results are more prone to noise. Therefore, an algorithm was developed that allows identification of the strongest interaction between the fragment and the target protein and to fix this interaction during cavity comparison (Figure S2). By using this approach, it is possible to enrich for cavities that possess the properties for forming a similar interaction.

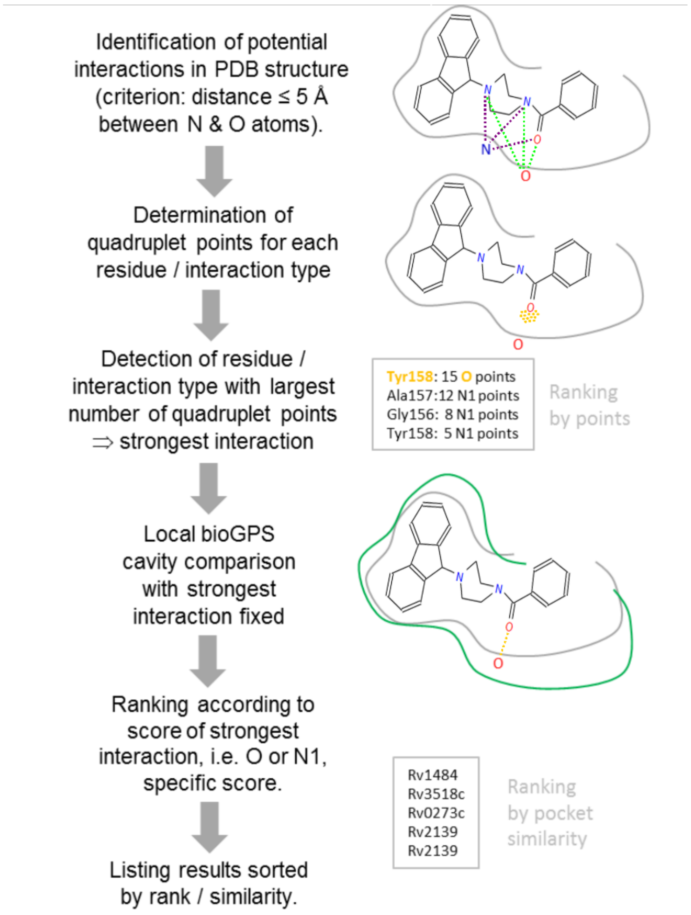

Figure S2. Schematic depiction of fragment-based BioGPS sub-pocket comparison.
First, all distances between nitrogen (N) and oxygen (O) atoms of the PDB ligand and the PDB protein are measured. For all O and N atoms of the protein that are within a distance of ≤ 5 Å of a N or O atom of the ligand, quadruplet points are calculated for the corresponding protein residues using the O or N1 probe, respectively. The residue for which the largest number of quadruplet points are found can be considered to form the strongest O | N1 interaction with the ligand. The binding site of the PDB structure is then compared to all sub-pockets of *M. tb.* structures keeping the strongest interaction fixed. Finally, the *M. tb.* structures are ranked according to the score of the strongest interaction. The *M. tb.* structures with binding sites that are most similar to the PDB ligand / fragment binding site can be found at the top of the ranking list.

D. Step 4: Docking of hit molecule

When the user has selected one of the structures with the most similar binding site among the structures in the *M. tb.* target space, the phenotypic active is docked into this potential target structure. This step should remove at least some of the false positives, when the full ligand does not bind, or the SAR does not fit with the binding pose. For this, the phenotypic active molecule is first transformed from a 2D into a 3D structure using LigPrep

[13] with the OPLS2005 force field [14]. In addition, formal charges are assigned with Epik [15,16] using a pH value of 7.4. Docking is performed with Glide [17-20] using default settings and allowing a maximum of 10 docking solutions per phenotypic active molecule.

In addition to standard docking using Glide default values, a docking with constraints can be performed. As the hit molecule and the ligand of the PDB structure used as template for cavity comparison contain the same fragment and the PDB structure possesses a binding site that is highly similar to the one of the identified target structure, it can be assumed that the respective fragment binds in the same way to both the PDB structure and the target structure. Therefore, interactions present between the fragment and the protein in the PDB structure can be set as constraints in docking of the whole phenotypic active molecule to the target structure. First, all hydrogen bonds between the PDB ligand fragment under consideration and the PDB protein structure are detected using a H bond acceptor distance ≤ 2.5 Å and a donor – H bond acceptor angle ≥ 120° as hydrogen bond criteria. These hydrogen bond interactions have been precomputed for PDB complex structures containing a ligand with at least one carbon atom, more than one nitrogen or oxygen atom and > 5 heavy atoms. The hydrogen bond information has been stored in a database table. For a particular PDB structure the information about donor or acceptor atoms is derived from the database and is stored in a list. Subsequently, the PDB structure and the target structure are super-positioned based on their binding sites using FLAP (BioGPS) [11,21]. For each atom of the PDB protein in the donor / acceptor list, a check is then made as to whether there is a donor / acceptor atom within 3.5 Å distance in the target structure. If this is the case, the corresponding atoms are defined as anchoring points for hydrogen bond constraints in Glide. For the ligand side of the docking constraints, SMARTS patterns defined based on the hydrogen bond information of the ligand side are used. By setting constraints in docking, the search space for the ligand position can be decreased and the hit molecule can be forced to adopt a binding mode that is similar to the binding mode found in the “template” PDB structure.

S2. Schematic depiction of workflow used for generating *M. tb.* models

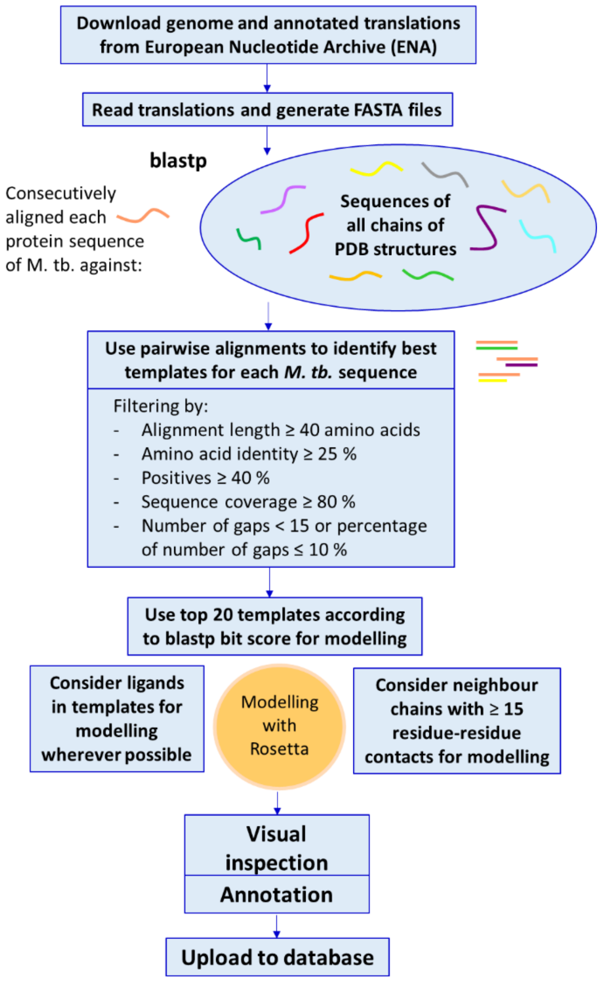

S3. The database

The backend of the platform consists of a MySQL database that contains PDB data, retrieved from http://files.rcsb.org and the ligands, fragments and fingerprints derived from the PDB files as well as *M. tb.* PDB and model structures. An overview of the main PDB associated data stored in the database is given in Table S1. Beside the PDB structures, the database contains tables storing the PDB ligands, all unique ligands present in the PDB, the hydrogen bonds in which the PDB ligands are involved, the PDB ligand fragments generated by *iChop++*, all unique fragments as well as the associated fingerprints and partial sums for scaffold hopping and sub-structure search.

Table S1. Overview of PDB associated database content	
Database content[a],[b]	No. of entries[c]
PDB structures	129,816
Ligands in PDB structures	779,453
Unique ligands present in PDB structures	24,031
Hydrogen bonds between PDB ligands and macromolecules	630,895
PDB ligand fragments generated by iChop++	5,801,041
Unique fragments with corresponding fingerprints and partial sums	595,951

[a] Data enabling primarily associations between the main data in the database are not mentioned. [b] Only data directly needed in the platform are listed. [c] Last update in May 2017

Table S2 gives an overview of the *M. tb.* structures stored in the database. There were 2,055 structures of proteins from *M. tb.* (May 2017) in the PDB. This represents 554 different proteins, some with ligands. Beside the 2,055 structures from the PDB we generated 3,667 model structures of *M. tb.* proteins with the aim of obtaining as many structures of *M. tb.* proteins as possible that could be modelled with high confidence. The 3,667 model structures represent 1,368 different *M. tb.* proteins. There are multiple models of some proteins because some represent models based on apo structures and some based on co-crystal structures with ligands. The ligands were imported into the modelled structures.

Table S2. Overview *M. tb.* associated database content	
Database content	No. of entries[a]
PDB structures of *M. tb.* proteins	2,055
Modelled structures of *M. tb.* proteins	3,667
Total number of *M. tb.* structures	5,722
Number of unique *M. tb.* proteins	1,602
Binding sites in *M. tb.* proteins	11,206

[a] Last update in May 2017.

S4. Pre-computed cavities and docking grids

In order to speed up the cavity comparison and docking steps, all the required files derived from the *M. tb.* structures were pre-computed. For *M. tb.* complex structures, binding sites were defined based on a distance of 5 Å (SubCav, FuzCav, and aCSM) / 2 Å (BioGPS) around the ligand / the ligand centered grid. For apo structures, first pockets were computed independently with FLAPsite [21] (default settings) and with SiteMap [22-24] (default parameters except dthresh = 3.0 Å). As the binding sites computed by flapsite are not evaluated for druggability, the three binding sites with the highest druggability score identified by SiteMap, were compared to the flapsite pockets. Among the flapsite pockets, those that had the largest number of pocket-defining points within 3.0 Å distance of the points defining the SiteMap pockets, were regarded as *apo* structure pockets with the best druggability. For computing the binding site cavities for the *apo* structures, the points defining the flapsite pockets were treated as the centers of the atoms of a ligand bound to a binding site. Cavities were computed as described for the protein-ligand complexes above. For each *M. tb.* structure input files for all cavity comparison approaches were pre-generated.

In the above described procedure cofactors that are in close contact with a ligand would normally be treated themselves as ligands and would be absent in the ligand cavity comparison. The BioGPS procedure is capable of handling the presence of cofactors during cavity comparison. First all cofactors with at least 3 atoms within a 4 Å distance of a ligand were identified. For those structures with close ligand-cofactor contacts, then binding site cavities were defined in which the cofactor was considered as part of the protein complex. If a complex contained two cofactors that fulfilled the distance criterion, alternative binding sites containing one or the other cofactor were created.

A python program enabling a fast computation of binding site cavities and docking grids for a large number of *M. tb.* structures has been prepared. Using this script it is straightforward to update the data stored on hard disc when new PDB structures become available and the database of *M. tb.* structures is updated.

S5. TIBLE

Table S3. Ligands from TIBLE database analysed in case study and corresponding targets as well as category of target prediction results according to Figure 4.

Ligand ChEMBL ID	Target	Target prediction results
CHEMBL1256993	GyrA, Rv0006	No similar PDB fragments/ligands
CHEMBL2017736	PknB, Rv0014c	Real prediction
CHEMBL1236228	Fba, Rv0363c	Self-search
CHEMBL519613	Def, Rv0429c	Almost self-search
CHEMBL1818383	Icl, Rv0467	DNA binding ligand
CHEMBL509952	FabH, Rv0533	Target not in database
CHEMBL2024344	MemB, Rv0548c	No similar PDB fragments/ligands
CHEMBL91	Cyp51, Rv0764c	Real prediction
CHEMBL1938813	Rv0948c	No similar PDB fragments/ligands
CHEMBL1762028	cfiB, Rv1106c	Reason for failure complex
CHEMBL238766	MshB, Rv1170	No similar PDB fragments/ligands
CHEMBL104	Cyp130, Rv1256c	Real prediction
CHEMBL1446150	MtcA1, Rv1284	Reason for failure complex
CHEMBL3115199	GlgE, Rv1327c	Reason for failure complex
CHEMBL1095991	RibH, Rv1416	Reason for failure complex
CHEMBL262343	InhA, Rv1484	Almost self-search
CHEMBL560315	ppnK, Rv1695	Real prediction
CHEMBL396982	scn, Rv1885c	No similar PDB fragments/ligands
CHEMBL459933	HisG, Rv2121c	No similar PDB fragments/ligands
CHEMBL1163074	mshC, Rv2130c	Real prediction
CHEMBL1651196	ftsZ, Rv2150c	No similar PDB fragments/ligands
CHEMBL1923646	mraY, Rv2156c	Target not in database
CHEMBL1765359	ptpA, Rv2234	No similar PDB fragments/ligands
CHEMBL399043	KasB, Rv2246	Almost self-search
CHEMBL106	Cyp121, Rv2276	Self-search
CHEMBL2418481	cysK1, Rv2334	Almost self-search
CHEMBL608841	mbtA, Rv2384	Reason for failure complex
CHEMBL116018	rpiB, Rv2465c	Self-search
CHEMBL567563	aroQ, Rv2537c	Almost self-search
CHEMBL2104121	dxs – Rv2682c	Target not in database

Table S3. continued.

Ligand ChEMBL ID	Target	Target prediction results
CHEMBL1823492	thyX, Rv2754c	Real prediction
CHEMBL64	folA, Rv2763c	Reason for failure complex
CHEMBL1823492	thyA, Rv2764c	Almost self-search
CHEMBL2431008	dxr, Rv2870c	Almost self-search
CHEMBL2259938	ligA, Rv3014c	No similar PDB fragments/ligands
CHEMBL2348058	nrdE, Rv3051c	No similar PDB fragments/ligands
CHEMBL571500	devR, Rv3133c	No similar PDB fragments/ligands
CHEMBL1928435	tmk, Rv3247c	Almost self-search
CHEMBL375328	rmlD, Rv3266c	Reason for failure complex
CHEMBL501294	Rv3273	No similar PDB fragments/ligands
CHEMBL3613833	birA - Rv3279c	Self-search
CHEMBL2048318	Cyp125, Rv3545c	Reason for failure complex
CHEMBL3222163	ispF, Rv3581c	No similar PDB fragments/ligands
CHEMBL20	mtcA2, Rv3588c	Reason for failure complex
CHEMBL428787	panC, Rv3602c	No similar PDB fragments/ligands
CHEMBL1644891	glfT2, Rv3808c	No similar PDB fragments/ligands
CHEMBL1235660	EthR, Rv3855	Self-search
CHEMBL2393579	trxB - Rv3913	No similar PDB fragments/ligands

References

[1] M. Congreve; R. Carr; C. Murray; H. Jhoti. A 'rule of three' for fragment-based lead discovery? Drug discovery today 8 (2003) 876-877.

[2] N.M. O'Boyle; M. Banck; C.A. James; C. Morley; T. Vandermeersch; G.R. Hutchison. Open Babel: An open chemical toolbox. Journal of cheminformatics 3 (2011) 33.

[3] K.T. Nguyen; L.C. Blum; R. van Deursen; J.L. Reymond. Classification of organic molecules by molecular quantum numbers. ChemMedChem 4 (2009) 1803-1805.

[4] P.E. Black. *Manhattan distance*, https://www.nist.gov/dads/HTML/manhattanDistance.html (2006).

[5] H.M. Berman; J. Westbrook; Z. Feng; G. Gilliland; T.N. Bhat; H. Weissig; I.N. Shindyalov; P.E. Bourne. The Protein Data Bank. Nucleic acids research 28 (2000) 235-242.

[6] K. Raman; K. Yeturu; N. Chandra. targetTB: a target identification pipeline for Mycobacterium tuberculosis through an interactome, reactome and genome-scale structural analysis. BMC systems biology 2 (2008) 109.

[7] Molsoft L.L.C., IcmJS (San Diego, California, 2017).

[8] T. Kalliokoski; T.S. Olsson; A. Vulpetti. Subpocket analysis method for fragment-based drug discovery. Journal of chemical information and modeling 53 (2013) 131-141.

[9] N. Weill; D. Rognan. Alignment-free ultra-high-throughput comparison of druggable protein-ligand binding sites. Journal of chemical information and modeling 50 (2010) 123-135.

[10] D.E. Pires; R.C. de Melo-Minardi; C.H. da Silveira; F.F. Campos; W. Meira, Jr. aCSM: noise-free graph-based signatures to large-scale receptor-based ligand prediction. Bioinformatics 29 (2013) 855-861.

[11] V. Ferrario; L. Siragusa; C. Ebert; M. Baroni; M. Foscato; G. Cruciani; L. Gardossi. BioGPS: Descriptors for Rational Engineering of Enzyme Promiscuity and Structure Based Bioinformatic Analysis. 9 (2014) e109354.

[12] L. Siragusa; S. Cross; M. Baroni; L. Goracci; G. Cruciani. BioGPS: navigating biological space to predict polypharmacology, off-targeting, and selectivity. Proteins 83 (2015) 517-532.

[13] Schrödinger Release 2016-4: LigPrep (Schrödinger, LLC, New York, NY., 2016).

[14] J.L. Banks; H.S. Beard; Y. Cao; A.E. Cho; W. Damm; R. Farid; A.K. Felts; T.A. Halgren; D.T. Mainz; J.R. Maple; R. Murphy; D.M. Philipp; M.P. Repasky; L.Y. Zhang; B.J. Berne; R.A. Friesner; E. Gallicchio; R.M. Levy. Integrated Modeling Program, Applied Chemical Theory (IMPACT). Journal of computational chemistry 26 (2005) 1752-1780.

[15] J.R. Greenwood; D. Calkins; A.P. Sullivan; J.C. Shelley. Towards the comprehensive, rapid, and accurate prediction of the favorable tautomeric states of drug-like molecules in aqueous solution. Journal of computer-aided molecular design 24 (2010) 591-604.

[16] J.C. Shelley; A. Cholleti; L.L. Frye; J.R. Greenwood; M.R. Timlin; M. Uchimaya. Epik: a software program for pK(a) prediction and protonation state generation for drug-like molecules. Journal of computer-aided molecular design 21 (2007) 681-691.

[17] Schrödinger Release 2016-4: Glide (Schrödinger, LLC, New York, NY, 2016).

[18] R.A. Friesner; J.L. Banks; R.B. Murphy; T.A. Halgren; J.J. Klicic; D.T. Mainz; M.P. Repasky; E.H. Knoll; M. Shelley; J.K. Perry; D.E. Shaw; P. Francis; P.S. Shenkin. Glide: a new approach for rapid, accurate docking and scoring. 1. Method and assessment of docking accuracy. Journal of medicinal chemistry 47 (2004) 1739-1749.

[19] R.A. Friesner; R.B. Murphy; M.P. Repasky; L.L. Frye; J.R. Greenwood; T.A. Halgren; P.C. Sanschagrin; D.T. Mainz. Extra precision glide: docking and scoring incorporating a model of hydrophobic enclosure for protein-ligand complexes. Journal of medicinal chemistry 49 (2006) 6177-6196.
[20] T.A. Halgren; R.B. Murphy; R.A. Friesner; H.S. Beard; L.L. Frye; W.T. Pollard; J.L. Banks. Glide: a new approach for rapid, accurate docking and scoring. 2. Enrichment factors in database screening. Journal of medicinal chemistry 47 (2004) 1750-1759.
[21] S. Cross; M. Baroni; E. Carosati; P. Benedetti; S. Clementi. FLAP: GRID Molecular Interaction Fields in Virtual Screening. Validation using the DUD Data Set. 50 (2010) 1442–1450.
[22] T.A. Halgren. Identifying and characterizing binding sites and assessing druggability. Journal of chemical information and modeling 49 (2009) 377-389.
[23] T. Halgren. New method for fast and accurate binding-site identification and analysis. Chemical biology & drug design 69 (2007) 146-148.
[24] Schrödinger Release 2016-4: SiteMap (Schrödinger, LLC, New York, NY, 2016).
